# Prognostic value of MRI-measured tumor thickness in patients with tongue squamous cell carcinoma

**DOI:** 10.1038/s41598-021-90655-z

**Published:** 2021-06-02

**Authors:** Ki-Sun Park, Yangsean Choi, Jiwoong Kim, Kook-Jin Ahn, Bum-soo Kim, Youn Soo Lee, Dong-Il Sun, Min-Sik Kim

**Affiliations:** 1 Department of Radiology, Seoul St. Mary’s Hospital, College of Medicine, The Catholic University of Korea, 222, Banpo-daero, Seocho-gu, Seoul, 06591 Republic of Korea; 2 Department of Hospital Pathology, Seoul St. Mary’s Hospital, College of Medicine, The Catholic University of Korea, 222, Banpo-daero, Seocho-gu, Seoul, 06591 Republic of Korea; 3 Department of Otolaryngology-Head and Neck Surgery, Seoul St. Mary’s Hospital, College of Medicine, The Catholic University of Korea, 222, Banpo-daero, Seocho-gu, Seoul, 06591 Republic of Korea

**Keywords:** Risk factors, Head and neck cancer, Oral cancer, Oral cancer detection

## Abstract

This study aimed to assess the prognostic value of MRI-measured tumor thickness (MRI-TT) in patients with tongue squamous cell carcinoma (SCC). This single-center retrospective cohort study included 133 pathologically confirmed tongue SCC patients between January 2009 and October 2019. MRI measurements of tongue SCC were based on axial and coronal T2-weighted (T2WI) and contrast-enhanced T1-weighted (CE-T1WI) images. Two radiologists independently measured MRI-TT. Intraclass correlation coefficients (ICC) were calculated for inter-rater agreements. Spearman’s rank correlation between MRI-TT and pathologic depth of invasion (pDOI) was assessed. Cox proportional hazards analyses on recurrence-free (RFS) and overall survival (OS) were performed for MRI-TT and pDOI. Kaplan–Meier survival curves were plotted with log-rank tests. The intra- and inter-rater agreements of MRI-TT were excellent (ICC: 0.829–0.897, all *P* < 0.001). The correlation between MRI-TT and pDOI was good (Spearman’s correlation coefficients: 0.72–0.76, *P* < 0.001). MRI-TT were significantly greater than pDOI in all axial and coronal T2WI and CE-T1WI (*P* < 0.001). In multivariate Cox proportional hazards analysis, MRI-TT measured on axial CE-T1WI yielded a significant prognostic value for OS (hazards ratio 2.77; *P* = 0.034). MRI-TT demonstrated excellent intra- and inter-rater agreements as well as high correlation with pDOI. MRI-TT may serve as a prognostic predictor in patients with tongue SCC.

## Introduction

Oral cavity squamous cell carcinoma (SCC) is the 8th most common cancer globally^1^ and oral tongue SCC is its most common subtype^2^. The prognostic significance of pathologic depth of invasion (pDOI) has been well-established^3–8^, such that pDOI was introduced in the recent 8th Edition of the American Joint Committee on Cancer (AJCC) and the Union for International Cancer Control (UICC), as a crucial part of T-staging in oral SCC^9–11^.

Prior to surgical treatment, preoperative MRI allows measurement of the tongue SCC thickness, which is often used for evaluating clinical T-stage. However, pDOI and MRI-measured tumor thickness (MRI-TT) are intrinsically different in that MRI-TT does not account for pathologic findings, such as epithelial involvement^12^. While the 8th Edition of the UICC/AJCC criteria proposed three classifications for the pDOI (≤ 5 mm, > 5 to ≤ 10 mm, and > 10 mm)^9–11^, there is no consensus regarding the reliable cutoff value of MRI-TT for prognostication as of yet.

pDOI is the gold standard reference that can only be determined postoperatively. A number of recent studies have investigated preoperative MRI for risk assessment in patients with tongue SCC^13–16^. Several studies have found that MRI-TT is an independent prognostic factor for predicting occult nodal metastasis^17–22^ and overall survival (OS)^23^ in oral cavity SCC. However, prognostic value of MRI-TT for recurrence-free survival (RFS) (i.e. free of local recurrence, lymph node metastasis, or distant metastasis) has not yet been investigated. Furthermore, defining the optimal MRI-TT measurement method deserves more research attention.

Addressing the prognostic value of MRI-TT, with respect to the gold standard pDOI, would aid in preoperative risk-stratification of patients. Therefore, this study aimed to determine the intra- and inter-rater agreement on various methods of measuring MRI-TT and to assess their performances for predicting RFS and OS in patients with tongue SCC.

## Results

### Patients and clinical profiles

**Figure 1:**
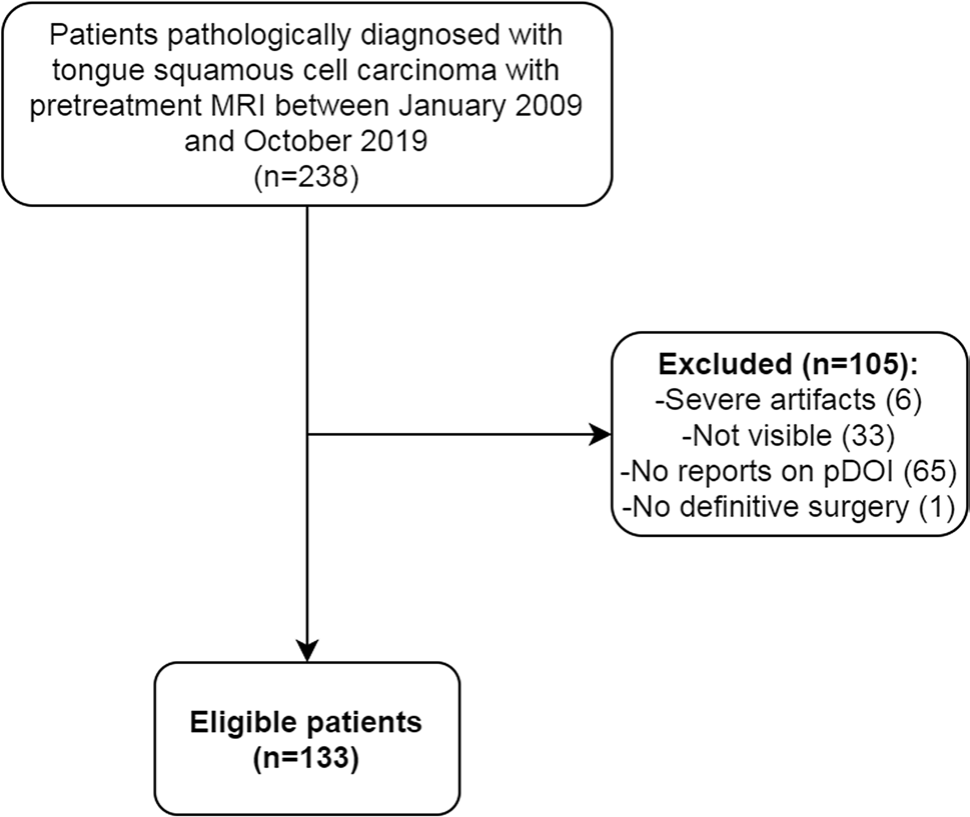
A flowchart presenting the patient selection process.

A total of 133 patients were retrospectively selected. The patient selection process is depicted in Fig. 1. The baseline characteristics of study cohort is summarized in Table 1. The mean age was 51 ± 15 years old [males, 77/133 (57.9%)]. There were 25 (18.8%) recurrences and 27 (20.3%) deaths during the follow-up period. The median RFS and OS were of 2.8 years (interquartile range 1.0–4.9 years) and 2.8 years (interquartile range 1.2–4.9 years), respectively. T-stage II had the most cases (44/133, 33.1%) followed by stage III (41/133, 30.8%), and stage I and IVa (both 24/133, 18.0%). N-stage 0 had the most cases (97/133, 72.9%) followed by stage II (25/133, 18.8%) and stage I (11/133, 8.3%). The median interval between biopsy and the MRI scan timing was 11 days (interquartile range 7–18 days). The median interval between MRI and the surgery was 12 days (interquartile range 7–20 days). The mean pDOI was 11.4 ± 9.2 mm; 20 patients (15%) had positive surgical margins in their pathologic specimens. Seventy‐five patients (56.4%) received surgery alone while 38 patients (28.6%) received both adjuvant chemotherapy and radiotherapy. Seventeen (12.8%) and three patients (2.3%) received adjuvant radiotherapy and chemotherapy, respectively.

**Table 1:** Baseline characteristics of study cohort.

Variables	n = 133
Age, mean ± SD	51 ± 15
Sex, male (%)	77 (57.9%)
**T stage**	
1	24 (18.0%)
2	44 (33.1%)
3	41 (30.8%)
4a	24 (18.0%)
**N stage**	
0	97 (72.9%)
1	11 (8.3%)
2	25 (18.8%)
3	0 (0)
Median interval between biopsy and MRI, days [interquartile range]	11 [7, 18]
Median interval between MRI and surgery, days [interquartile range]	12 [7, 20]
**Surgery**	
Wide excision	5 (3.8%)
Partial glossectomy	93 (69.9%)
Hemiglossectomy	13 (9.8%)
Subtotal glossectomy	3 (2.3%)
Near total glossectomy	7 (5.3%)
Total glossectomy	12 (9.0%)
Adjuvant chemotherapy, n (%)	41 (30.8%)
Adjuvant radiation, n (%)	55 (41.4%)
pDOI (mm), mean ± SD	11.4 ± 9.2
Positive surgical margin, n (%)	20 (15.0%)
Median overall survival, years [interquartile range]	2.8 [1.2, 4.9]
Death, n (%)	27 (20.3%)
Median recurrence-free survival, years [interquartile range]	2.8 [1.0, 4.9]
**Recur, n (%)**	25 (18.8%)
Local recurrence	14 (10.5%)
Lymph node metastasis	4 (3%)
Distant metastasis	2 (1.5%)
Local recurrence and lymph node metastasis	3 (2.2%)
Local recurrence and distant metastasis	1 (0.8%)
Local recurrence, lymph node and distant metastasis	1 (0.8%)

*pDOI* pathologic depth of invasion, *SD* standard deviation.

### MRI-TT measurements and agreement with pDOI

**Figure 2:**
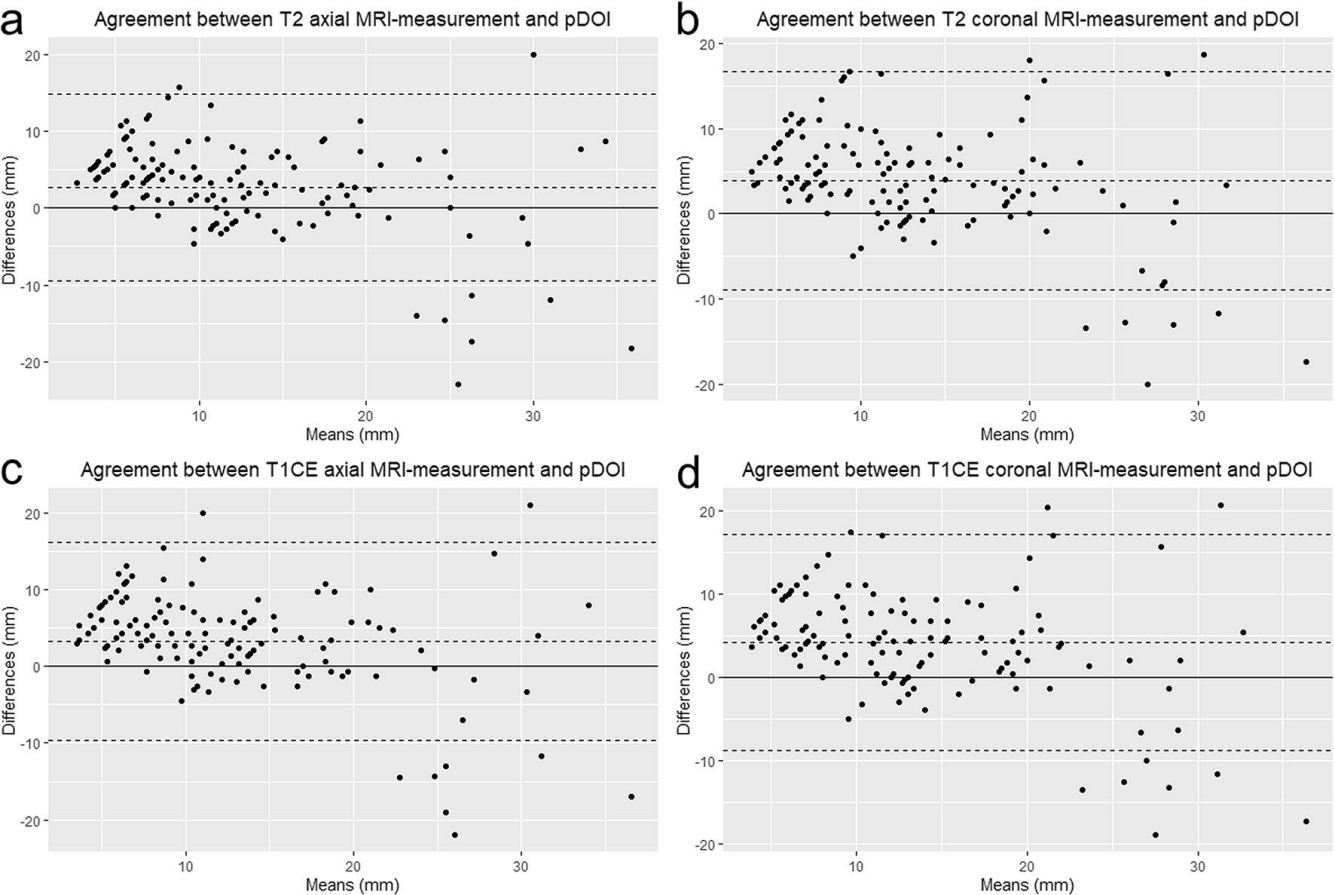
Bland–Altman plots illustrating the absolute differences between MRI-measured tumor thickness (MRI-TT) measured on (**a**) axial T2WI, (**b**) coronal T2WI, (**c**) axial CE-T1WI, (**d**) coronal CE-T1WI, and pathologic depth of invasion (pDOI). The central dotted horizontal line represents the mean difference whereas the upper and lower dotted horizontal lines represent the upper and lower 95% confidence intervals of agreement, respectively.

**Figure 3:**
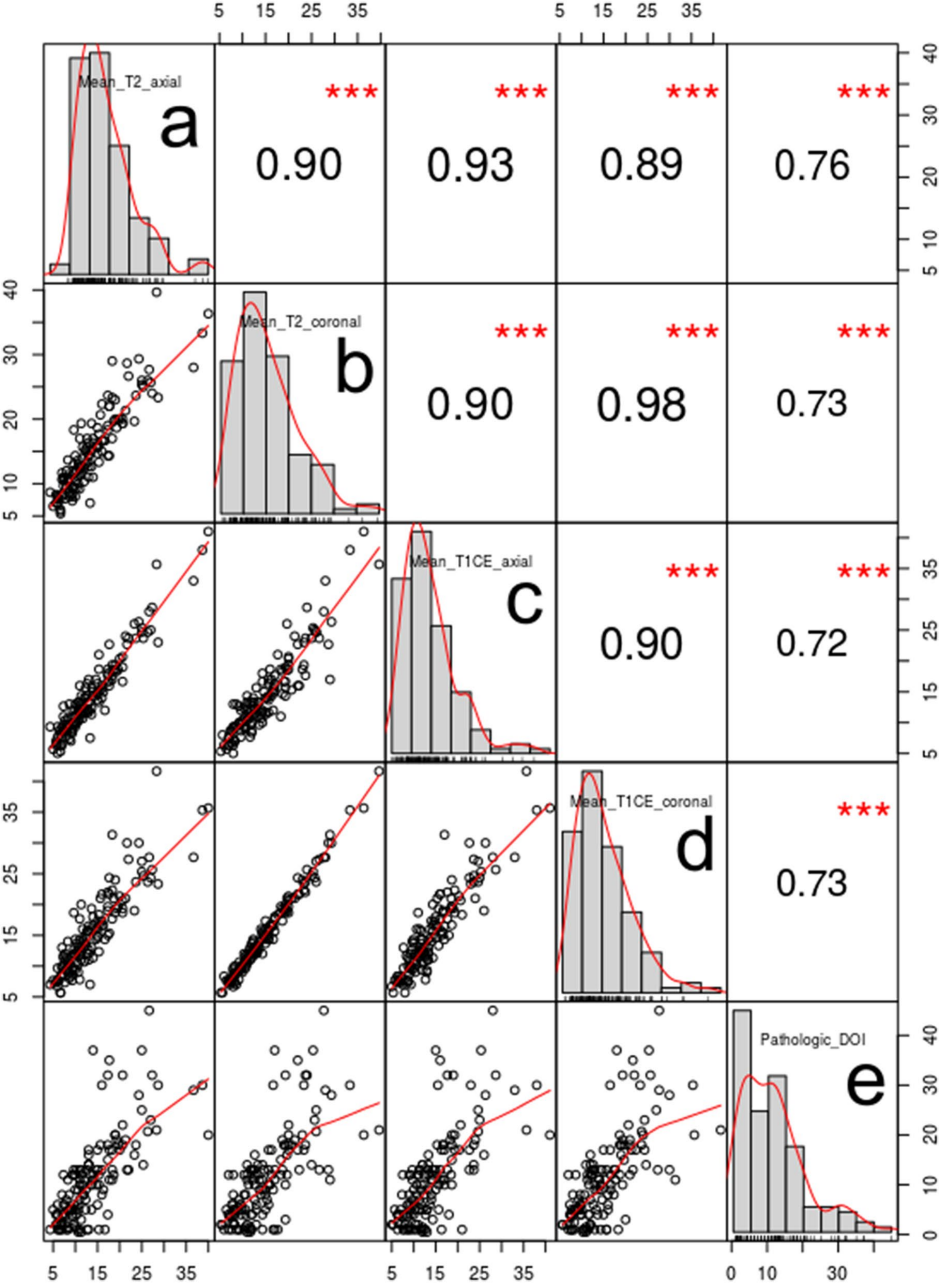
Correlation matrix of MRI-measured tumor thickness (MRI-TT), (**a**) axial T2WI, (**b**) coronal T2WI, (**c**) axial CE-T1WI, (**d**) coronal CE-T1WI, and (**e**) pathologic depth of invasion (pDOI). The numbers on the x- and y-axes indicate MRI-TT or pDOI in millimeters. Spearman rank correlation coefficients are shown with three stars indicating *P* < 0.001 (top right). Bivariate scatterplots are shown with fitted lines (bottom left).

The intra- and inter-rater agreements for all the MRI measurements were excellent (ICC: 0.829–0.858 and 0.853–0.897, respectively; all *P* < 0.001) (Table 2(a)). In comparing the absolute values between MRI-TT and pDOI, all MRI measurements showed significantly greater tumor thicknesses than pDOI (*P* < 0.001) (Table 2(b)), with axial measurements showing lesser differences than coronal measurements (2.66–3.24 mm vs. 3.93–4.10 mm). The agreements between MRI-TT and pDOI were good overall with ICC ranging from 0.624 to 0.731 (Table 2(c)). The Bland–Altman plots also showed good agreements between MRI-TT and pDOI (Fig. 2). The corr elation matrix between MRI-TT and pDOI is illustrated in Fig. 3. The correlation coefficients were high between MRI-TT and pDOI (0.72–0.76, all *P* < 0.001) and were higher among the different methods of MRI-TT (0.89–0.93, all *P* < 0.001).

**Table 2:** Agreement and comparison of MRI-measured tumor thickness and pathologic DOI.

(a) Intra- and interrater agreement among MRI-measured DOI					
Measurements	Rater 1a	Rater 1b	Intra-rater agreement	Rater 2*	Inter-rater agreement
T2 axial (mm), median [IQR]	13 [9.0–17.0]	13 [9.0–18.0]	0.858 [0.806–0.897]	13.0 [9.0–17.0]	0.885 [0.842–0.917]
T2 coronal (mm), median [IQR]	14.0 [10.0–18.0]	14 [10.0–19.3]	0.829 [0.766–0.876]	14.0 [10.0–19.0]	0.853 [0.799–0.894]
T1CE axial (mm), median [IQR]	13.0 [9.0–17.0]	13.0 [10.0–18.0]	0.858 [0.805–0.898]	13.0 [10.0–18.0]	0.866 [0.814–0.904]
T1CE coronal (mm), median [IQR]	13.0 [10.0–19.0]	14.0 [11.0–20.3]	0.846 [0.788–0.889]	14.0 [10.0–18.0]	0.897 [0.857–0.926]

(b) Comparison between radiologic and pathological measurements of DOI					
MRI measurement	Mean tumor thickness (mm)	pathologic DOI (mm)	Mean difference (mm)	95% CI	P-value
T2WI axial	14.0 ± 6.9	11.4 ± 9.2	2.66	1.59–3.73	< 0.001
T2WI coronal	15.3 ± 6.7		3.93	2.82–5.05	< 0.001
T1CE axial	14.6 ± 6.7		3.24	2.10–4.37	< 0.001
T1CE coronal	15.5 ± 6.8		4.1	2.96–5.23	< 0.001

(c) Agreement between MRI-measured tumor thickness and pathologic DOI					
Measurements	Intraclass correlation coefficient (95% CI)				
	Rater 1a	Rater 1b	Rater 2		
T2 axial	0.629 (0.514–0.722)	0.731 (0.641–0.801)	0.700 (0.601–0.777)		
T1CE axial	0.628 (0.511–0.722)	0.630 (0.515–0.723)	0.681 (0.578–0.762)		
T2 coronal	0.662 (0.553–0.748)	0.713 (0.618–0.788)	0.663 (0.555–0.749)		
T1CE coronal	0.671 (0.564–0.756)	0.624 (0.507–0.718)	0.658 (0.549–0.744)		

All P-values were < 0.001.

*Agreement with Rater 1a; all P-values were < 0.001.

### Cox proportional hazards analysis on recurrence-free survival

**Figure 4:**
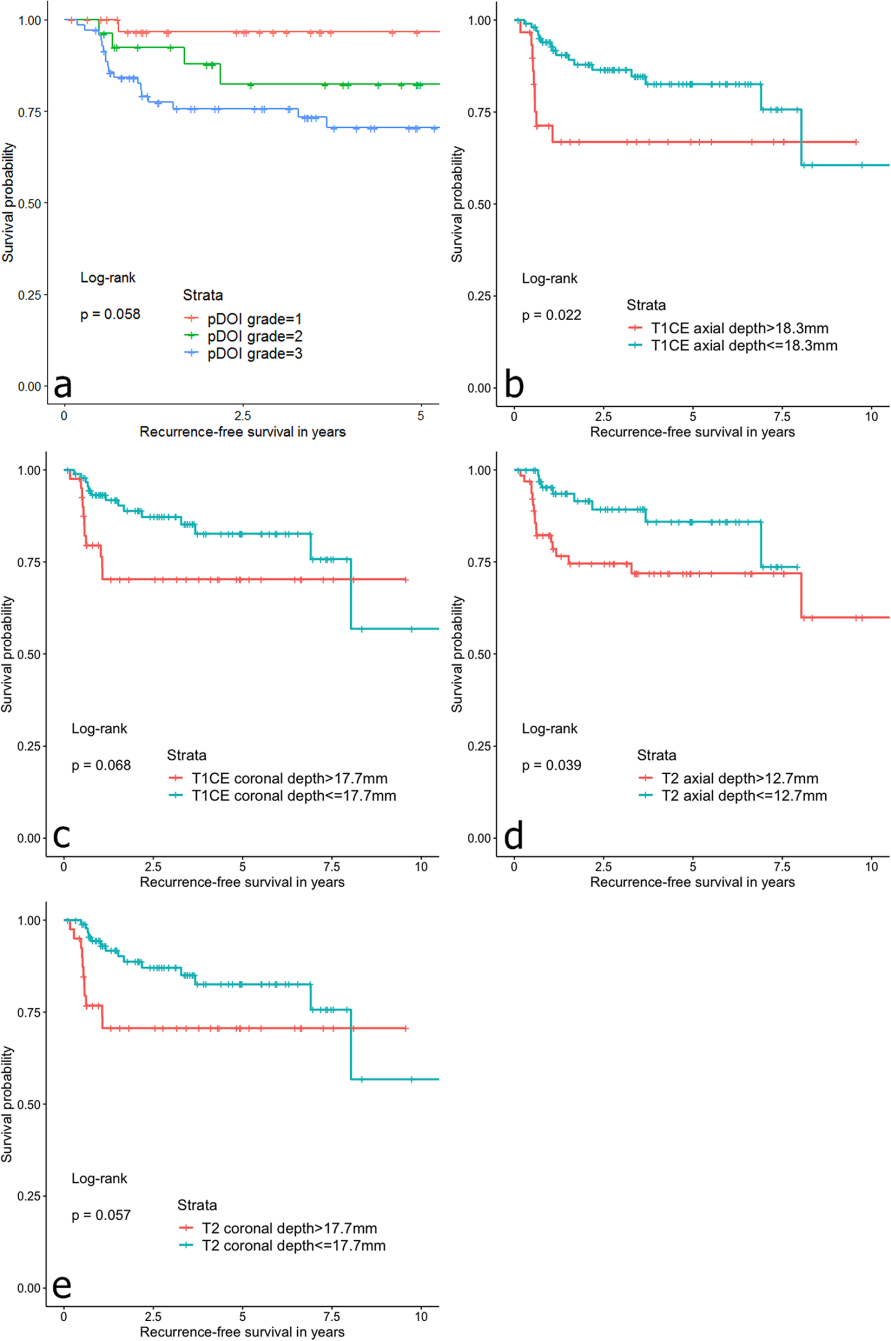
Kaplan–Meier curves for recurrence-free survivals based on (**a**) pathologic depth of invasion (pDOI) grades, (**b**) axial CE-T1WI, (**c**) coronal CE-T1WI, (**d**) axial T2WI, and (**e**) coronal T2WI, stratified by the thicknesses indicated.

The hazards ratios and 95% confidence intervals for RFS of pDOI and MRI-TT, stratified by respective thicknesses, are summarized in Table 3. T2WI axial thickness of greater than 12.7 mm was significantly associated with a greater risk of recurrence (HR 2.38; *P* = 0.046). CE-T1WI axial thickness of greater than 18.3 mm was associated with a greater risk of recurrence (HR = 2.34; *P* = 0.057). pDOI grade was significantly associated with greater risks of recurrences (HR 2.06; *P* = 0.024). Coronal measurements of MRI-TT predicted recurrences, but without statistical significances (T2WI coronal > 17.7 mm; HR 2.12; *P* = 0.063 and CE-T1WI coronal > 17.7 mm; HR 2.12; *P* = 0.063). Among the clinical variables, age was significantly associated with RFS (HR 1.03; *P* = 0.022). In multivariate analysis including age, axial T2WI, and pDOI grade, age remained as a significant variable (HR 1.03; *P* = 0.046). The Kaplan–Meier curves of MRI-TT and pDOI demonstrated similar results (Fig. 4).

**Table 3:** Cox proportional hazards analysis of recurrence-free survival.

(a) Univariate analysis				(b) Multivariate analysis*			
Clinical variable	HR	95% CI	*P*	Variable	HR	95% CI	*P*
Sex, male	1.32	0.57–3.07	0.522				
Age	1.03	1.01–1.06	0.022	Age	1.03	1.00–1.06	0.046
T-stage, T1/T2	0.43	0.19–1.0	0.050				
N-stage, N0	1.13	0.45–2.82	0.802				
Surgical margin, positive	0.67	0.20–2.26	0.522				
DOI variable**							
T2WI axial, > 12.7 mm	2.38	1.02–5.56	0.046	T2WI axial, > 12.7 mm	1.23	0.43–3.52	0.694
T2WI coronal, > 17.7 mm	2.12	0.96–4.67	0.063				
T1CE axial, > 18.3 mm	2.34	0.98–5.62	0.057				
T1CE coronal, > 17.7 mm	2.12	0.96–4.67	0.063				
pDOI grade***	2.06	1.10–3.87	0.024	pDOI grade	1.81	0.84–3.91	0.131

*HR* hazards ratio, *CI* confidence interval, *T2WI* T2-weighted image, *T1CE* T1-weighted contrast enhanced image, *pDOI* pathologic depth of invasion.

*Variables with *P* < 0.05 from univariate analysis were chosen.

**Cutoff values were determined by optimal stratification based on log-rank statistics.

***Grade 1 (pDOI < 5 mm); grade 2 (pDOI 5–10 mm); grade 3 (pDOI ≥ 10 mm).

### Cox proportional hazards analysis on overall survival

**Figure 5:**
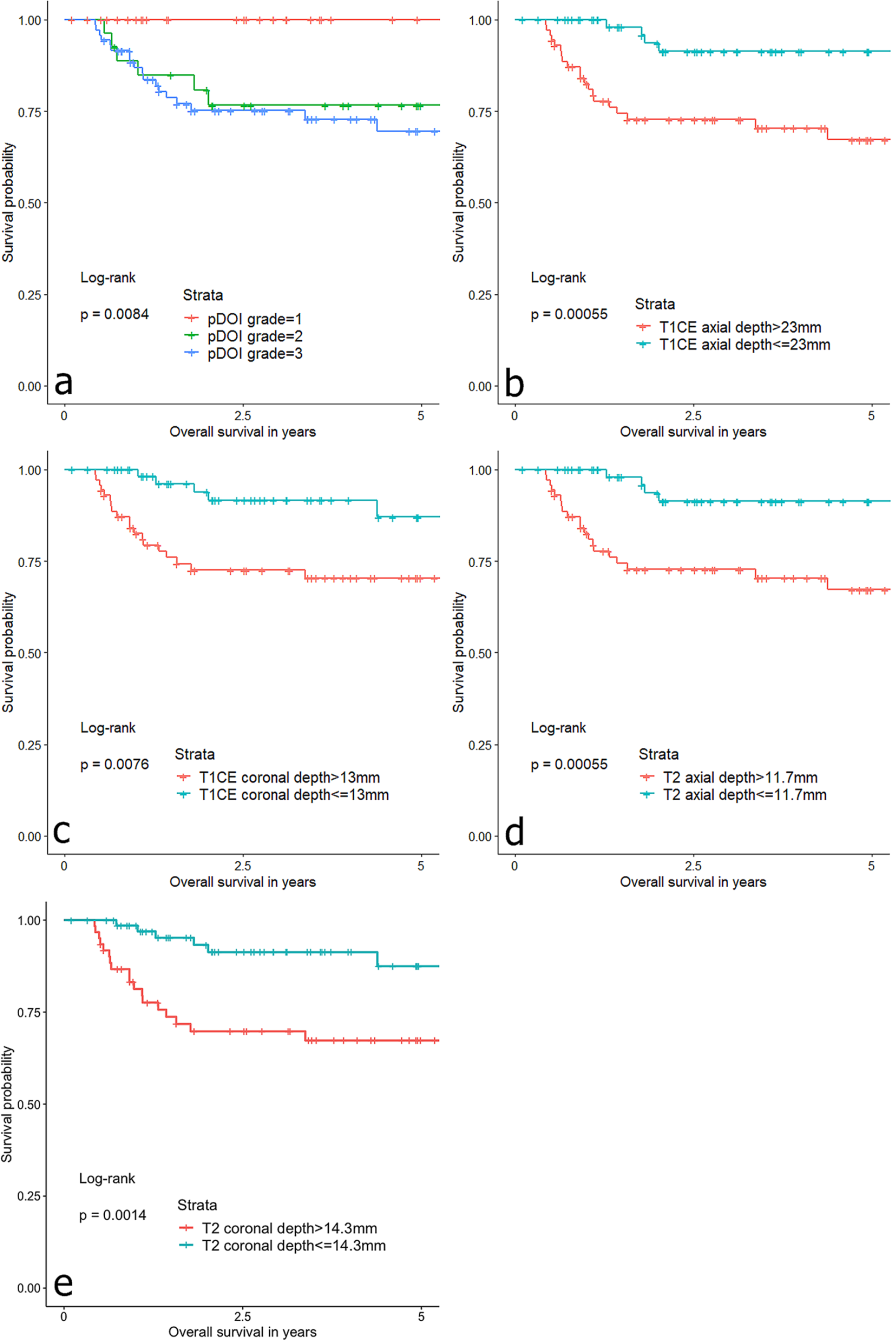
Kaplan–Meier curves for overall survivals based on (**a**) pathologic depth of invasion (pDOI) grades, (**b**) axial CE-T1WI, (**c**) coronal CE-T1WI, (**d**) axial T2WI, and (**e**) coronal T2WI, stratified by the thicknesses indicated.

The hazards ratios and 95% confidence intervals for OS are summarized in Table 4. T2WI and CE-T1WI axial thickness of greater than 11.7 mm and 23 mm, respectively, were significantly associated with worse prognosis (HR 5.34; *P* = 0.002 and HR 5.03; *P* < 0.001, respectively). pDOI grade was significantly associated with worse prognosis (HR 2.73; *P* = 0.006). Coronal measurements of MRI-TT demonstrated significances in predicting OS (T2WI coronal > 14.3 mm; HR 3.10; *P* = 0.01 and CE-T1WI coronal > 13 mm; HR 3.23; *P* = 0.012). Among the clinical variables, age and T-stage of T1/T2 were significantly associated with OS (HR 1.03; *P* = 0.023 and HR 0.33; *P* = 0.012, respectively). The Kaplan–Meier curves of MRI-TT and pDOI demonstrated similar results (Fig. 5). In multivariate analysis including age, T-stage, axial T2WI, coronal T2WI, axial CE-T1WI, and pDOI grade, CE-T1WI axial > 23 mm remained significant (HR 2.77; *P* = 0.034).

**Table 4:** Cox proportional hazards analysis of overall survival.

(a) Univariate analysis				(b) Multivariate analysis*			
Clinical variable	HR	95% CI	*P*	Variable	HR	95% CI	*P*
Sex, male	1.226	0.57–2.81	0.572				
Age	1.03	1.00–1.06	0.023	Age	1.02	1.00–1.05	0.111
T-stage, T1 or T2	0.33	0.14–0.78	0.012	T-stage, T1 or T2	1.43	0.41–4.95	0.576
N-stage, N0	0.51	0.24–1.1	0.087				
Surgical margin, positive	0.60	0.18–2.0	0.405				
DOI variable**							
T2WI axial, > 11.7 mm	5.34	1.84–15.5	0.002	T2WI axial, > 11.7 mm	2.56	0.54–11.7	0.235
T2WI coronal, > 14.3 mm	3.10	1.31–7.34	0.01	T2WI coronal, > 14.3 mm	0.69	0.10–4.78	0.711
T1CE axial, > 23 mm	5.03	2.17–11.6	< 0.001	T1CE axial, > 23 mm	2.77	1.08–7.12	0.034
T1CE coronal, > 13 mm	3.23	1.3–8.0	0.012	T1CE coronal, > 13 mm	1.61	0.23–11.3	0.631
pDOI grade***	2.73	1.33–5.62	0.006	pDOI grade	1.77	0.21–2.55	0.282

*HR* hazards ratio, *CI* confidence interval, *T2WI* T2-weighted image, *T1CE* T1-weighted contrast enhanced image, *pDOI* pathologic depth of invasion.

*Variables with *P* < 0.05 from univariate analysis were chosen.

**Cutoff values were determined by optimal stratification based on log-rank statistics.

***Grade 1 (pDOI < 5 mm); grade 2 (pDOI 5–10 mm); grade 3 (pDOI ≥ 10 mm).

## Discussion

The current study demonstrated excellent intra- and inter-rater reliabilities of MRI-TT, which also showed high correlation with pDOI. In multivariate analysis, axial CE-T1WI of > 23 mm demonstrated statistical significance with respect to OS while pDOI grade did not. The results of this study suggest the potential use of MRI-TT in preoperative risk-stratification of tongue SCC patients.

A previous study by Mao et al.^15^ reported the prognostic MRI depths of tongue SCC that enabled the prediction of overall and disease-specific survival as 11 mm, which is similar to our cutoff value of 11.7 mm determined on axial T2WI. Their image analysis used pathologic specimen as reference template for measurement, allowing MRI measurement much closer to the pDOI. Our findings based solely on MRI measurements might provide a more practical prerequisite for risk-stratification of patients preoperatively.

Our reported mean difference between MRI-TT and pDOI of 2.7–4.1 mm was similar to a study by Yesuratnam et al.^24^ who showed a mean difference of 3.2 ± 4.9 mm on T2WI and 3.0 ± 4.4 mm on CE-T1WI. However, their study did not specify whether the measurements were based on either axial or coronal MRI. The current study demonstrated that axial measurements on both T2WI and CE-T1WI have smaller mean differences compared to the coronal measurements. This finding could further shed light on choosing the optimal reconstruction method of MRI in determining MRI-TT.

Several similar studies included patients with limited T stages^5,13,16,20,25^, whereas the current study included a large sample size of patients with various T and N stages. This strengthens our findings to be more applicable in a wider range of disease severity for patients with tongue SCC. Another strength of this study includes the image analysis based on multi-parametric (T2 and CE-T1WI) and multi-reconstructed MRI (axial and coronal), providing four different measurement methods. A similar study by Murakami et al.^13^ also employed three different methods of measurement on MRI; however, in addition to the larger sample size of our cohort, the survival analysis of this study yielded significant prognostic values derived from various measurement methods, potentially providing the optimal method for prognostication.

While MRI is the imaging modality of choice for assessing tongue SCC, the discrepancy between MRI-TT and pDOI, such as overestimated values of MRI-TT, has been reported in previous literature^13,15,24,26^. One possible explanation is due to shrinkage of specimens after formalin fixation. In a study by Chen et al.^27^, head and neck cancer specimens’ length, width and depth shrank on average by 4.4% to 6.2% after formalin fixation. However, Umstattd et al.^28^ found that shrinkage of mucosal margins of oral cavity squamous cell carcinoma specimens occurred mostly between pre-excision and post-excision—prior to formalin fixation, thus suggesting that the shrinkage might be due to intrinsic tissue properties. With the presented data, it is difficult to determine whether the shrinkage occurred immediately after excision or due to formalin-fixation. However, understanding the inherent discrepancy between MRI-TT and pDOI would allow more informed clinical decision making. One interesting side finding was that larger tongue SCC tended to demonstrate weaker correlation between MRI-TT and pDOI, as evidenced in both Bland–Altman and correlation matrix plots. This finding suggests that larger tongue SCC would require more cautious interpretation.

Of importance, MRI-TT seemed to show more prognostic value for predicting OS than RFS. In univariate Cox proportional hazards analysis, all four different methods of MRI-TT were found to be significantly associated with OS, whereas one method—axial T2WI—was significantly associated with RFS. In multivariate analysis, only MRI-TT over 23 mm on axial CE-T1WI was found to be independently associated with OS. These weak associations in multivariate results suggest that MRI-TT should be interpreted as a complimentary measure along with other factors.

There are several limitations of the study that must be addressed. First, in addition to the retrospective study design, 20 patients had positive surgical margins in their pathologic specimens. However, other than that, the patients were included according to the predefined strict selection criteria, which would have minimized the possibility of selection bias. Second, the standard protocol in our institution employed MRI slice thickness of 4 mm, which could have missed very thin SCC that might have been present. Third, the number of recurrences was small (n = 25) to obtain optimal accuracy of regression coefficients. Studies with a larger number of cases of recurrence and deaths are warranted for precise multivariate survival modelling. Finally, the potential impact of pre-MRI biopsy was not accounted for; the biopsy might have increased the extent of tumor enhancement, possibly yielding overestimated measurements.

In conclusion, MRI-TT demonstrated excellent intra- and inter-rater agreements and high correlation with pDOI. MRI-TT above certain threshold thicknesses showed prognostic value in patients with tongue SCC.

## Materials and methods

### Ethical consideration

This single-center retrospective cohort study was approved by the Institutional Review Board of Seoul St. Mary’s Hospital, Seoul, Republic of Korea (KC20RISI0216). Written consent for medical research was waived by the Institutional Review Board of Seoul St. Mary’s Hospital due to the retrospective nature of study. All methods were performed in accordance with the ethical standards of our institutional research committee and with the 1964 Helskinki declaration and its later amendments.

### Patients

The records of consecutive patients diagnosed with tongue SCC at our institution from January 2009 to October 2019 were retrospectively reviewed. The inclusion criteria of the patients were: (1) pathologically diagnosed with tongue SCC and underwent surgery; (2) has available pretreatment MRI with sequences, including axial and coronal T2-weighted images (T2WI) and contrast-enhanced T1-weighted images (CE-T1WI); and (3) does not have distant metastasis at presentation. The exclusion criteria were as follows: (1) prior treatment history; (2) severe artifacts affecting appropriate image analysis; (3) tongue SCC not visible on the MRI scans; and (4) no pDOI report.

### Clinical profiles

The following clinical factors were reviewed from the electronic medical records of our institution: age at diagnosis, sex, type of surgery (i.e. wide excision, partial, hemi, near total, or total glossectomy), history of adjuvant chemotherapy and radiation therapy, and pathologic reports. All patients were assigned a pathological TNM stage according to the 8th Edition of the AJCC/UICC staging system^9–11^. All patients were treated with definitive primary tumor resection and neck dissection. Location of unilateral neck dissection was determined based on the location of primary cancer (i.e. left or right); bilateral modified radical neck dissection was performed for patients with metastatic cervical lymph node at presentation. Indications for adjuvant radiotherapy or chemotherapy were variable but were primarily based on the following findings: positive or close margins found on the resection, advanced T classification, lymphovascular or perineural invasion, multiple nodal metastasis, or extracapsular spread. The tissue samples from resected specimens were formalin-fixed for at least 7 h and embedded in paraffin. Representative tissue areas were stained with hematoxylin and eosin. The pDOI was measured on paraffin-embedded sections by an experienced head and neck pathologist with 20 years of experience. The pDOI was measured as the perpendicular distance from the mucosal surface of tumor to the deepest point of invasion in any plane.

The cutoff values of pDOI to stratify the patients into low-risk or high-risk SCC were 5 mm and 10 mm, respectively, as proposed by the 8th Edition of the AJCC/UICC criteria^9–11^. RFS was defined as the interval between the date of pathological confirmation of SCC and the date of pathologically confirmed recurrence (i.e. either local recurrence, lymph node metastasis, or distant metastasis), last clinical follow-up, or death. Similarly, OS was defined as the interval between the date of diagnosis and the date of death or last clinical follow-up. Patients underwent MRI scans at 6-month intervals during the first two years following the initial surgery, after which MRI was scanned annually. The mean clinical follow-up was 3.4 years (range 0.1–10.7 years) and ended on February 18, 2020.

### Image acquisition

All MRI were acquired from a 3.0 Tesla scanner (Verio, Siemens Healthineers, Erlangen, Germany) with a 12-channel head coil. The CE-T1WI were acquired with gadolinium-based contrast agent (0.1 mmol/kg, Gadovist, Bayer, Germany). The acquisition parameters were: (a) axial T2WI; repetition time/echo time, 3600/87 ms; flip angle, 150°; matrix, 320 × 256; field of view, 200 × 200 mm^2^; slice thickness, 4 mm; (b) coronal T2WI; repetition time/echo time, 4760/81 ms; matrix, 320 × 256; field of view, 220 × 220 mm^2^; slice thickness, 4 mm; (c) axial CE-T1WI; repetition time/echo time, 610/16 ms; flip angle, 150°; matrix, 320 × 256; field of view, 200 × 200 mm^2^; slice thickness, 4 mm; and (d) coronal CE-T1WI; repetition time/echo time, 693/14 ms; flip angle, 150°, matrix, 320 × 250; field of view, 220 × 220 mm^2^; slice thickness, 4 mm.

### Image analysis

**Figure 6:**
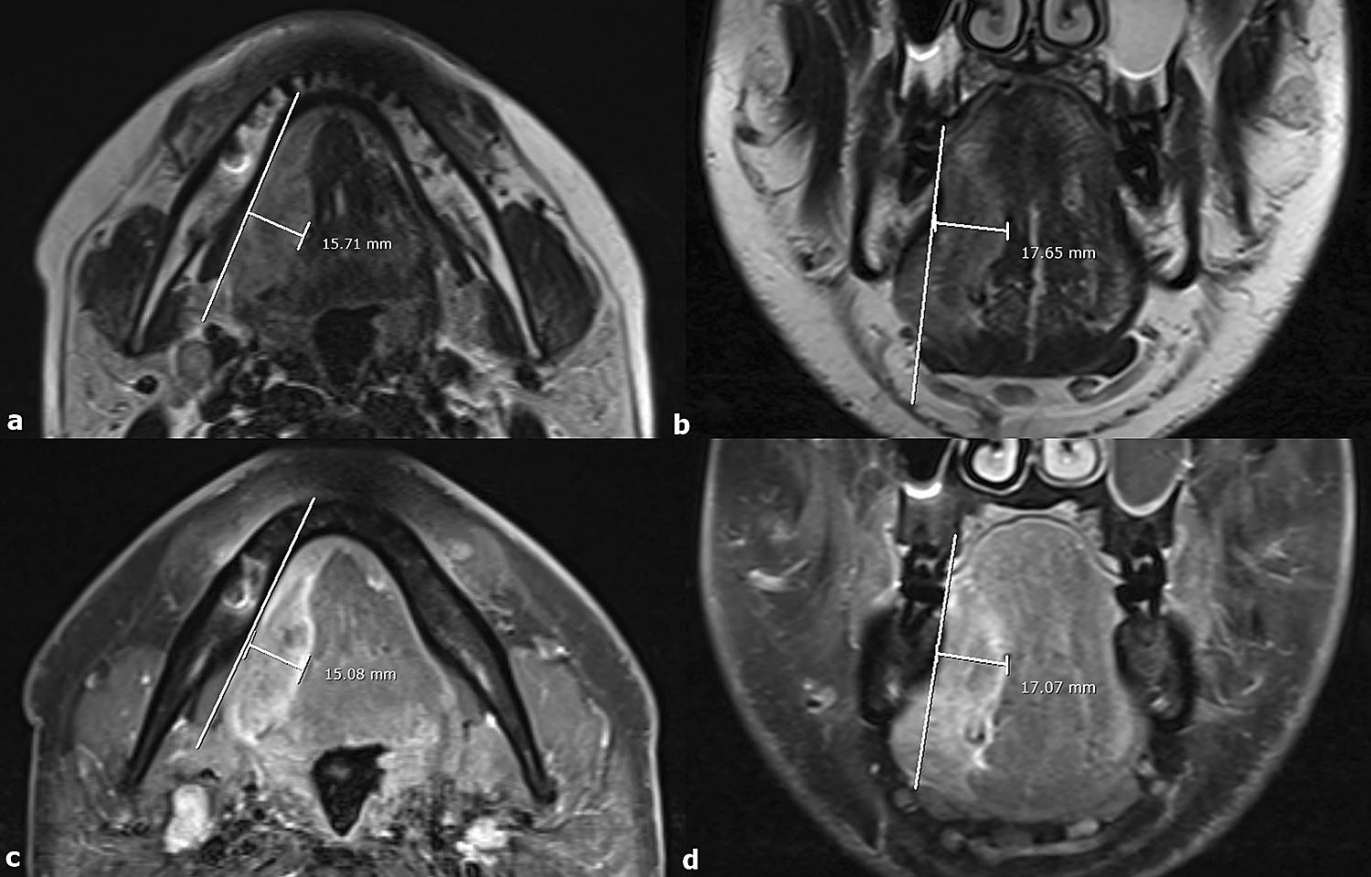
Representative measurements of MRI-measured tumor thickness (MRI-TT) on (**a**) axial T2WI, (**b**) coronal T2WI, (**c**) axial CE-T1WI with fat suppression, and (**d**) coronal CE-T1WI with fat suppression in a 39-year-old male patient diagnosed with tongue squamous cell carcinoma.

The two raters with 3 and 8 years of experience in head and neck imaging independently analyzed all the images. To minimize recall bias, the first rater repeated the analysis after three months. Both raters were blinded to the patients’ clinical information during image analysis. Two different MR sequences (T2WI and CE-T1WI) with axial and coronal acquisitions were used to measure the tumor thickness. For both axial and coronal scans, the reference line was drawn along the tumor’s lateral mucosal margin at the most representative image where the tumor appeared largest. A line perpendicular to the reference was drawn to the deepest point and was determined as MRI-TT. All measurements were performed using our in-house picture archiving communication system software. Representative images of the MRI-TT measurements are illustrated in Fig. 6.

### Statistical analysis

The normality of the MRI-TT and pDOI measurements was tested via Shapiro–Wilk test. The continuous variables were reported as the mean and standard deviation or median and interquartile range, as appropriate. For categorical variables, frequencies and proportions were reported. The intra- and inter-rater reliabilities were evaluated via intraclass correlation coefficients (ICC) with two-way random effects models. Bland–Altman plots^29^ were plotted for visual assessment of agreements between mean MRI-TT and pDOI. The difference between MRI-TT and pDOI was assessed via paired Wilcoxon signed-rank test. Spearman rank correlation coefficients were calculated and visualized via correlation matrix of the different methods of MRI-TT and between MRI-TT and pDOI. Survival analyses on RFS and OS was performed via Cox proportional hazards models. Kaplan–Meier curves were plotted with log-rank tests to compare the survival curves. The cutoff values for pDOI were grouped into grade 1 (< 5 mm), grade 2 (5–10 mm), and grade 3 (> 10 mm) based on the 8th edition of the AJCC/UICC^11^, while those for MRI-TT were determined by optimal stratification based on log-rank statistics^30^. All statistical analyses were performed using R statistical software (v. 3.6.1, R Foundation for Statistical Computing, Vienna, Austria, 2019, https://www.R-project.org). Statistical significance was set at *P* < 0.05.
